# Hepatitis B: A Current Global Health Dilemma

**Published:** 2014-01-22

**Authors:** Jessica Howell, Nimzing G Ladep, Maud Lemoine, Mark R Thursz, Simon D Taylor-Robinson

**Affiliations:** 1 Department of Medicine, Imperial College London, St Mary’s Campus, London, United Kingdom; Centre for Population Health, Macfarlane-Burnet Institute, Prahran, Victoria, Australia; Department of Medicine, University of Melbourne, Parkville, Victoria, Australia; 2 Department of Medicine, Imperial College London, St Mary’s Campus, London, United Kingdom; Department of Medicine, Jos University Teaching Hospital, Plateau State, Nigeria; 3 Department of Medicine, Imperial College London, St Mary’s Campus, London, United Kingdom; Medical Research Council (The Gambia Unit), Fajara, Banjul, The Gambia; 4 Department of Medicine, Imperial College London, St Mary’s Campus, London, United Kingdom; 5 Department of Medicine, Imperial College London, St Mary’s Campus, London, United Kingdom

Hepatitis B virus (HBV) infection causes a spectrum of acute and chronic liver disease, with chronic infection ranging from inactive carrier status to progressive chronic hepatitis, culminating in end-stage cirrhosis and liver cancer. Over one-third of the world’s population has been or is currently infected with HBV and 350 to 400 million people remain chronic hepatitis B surface antigen (HBsAg) carriers.^1^ There are over 500 to 750,000 deaths annually due to HBV-related cirrhosis and liver cancer worldwide, with this figure likely to be underestimated due to inadequate disease and cancer surveillance in many resource-poor countries, where HBV is endemic^1^ [World Health Organization (WHO). Hepatitis B (online). Available at: http://www.who.int/csr/disease/hepatitis/hepatitisB_whocdscsrlyo2002_2.pdf. Accessed on: 21st April 2014].

**Figure:**
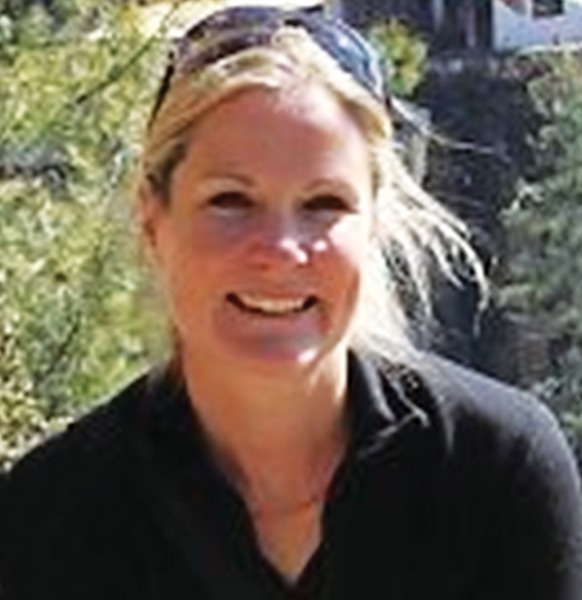
Jessica Howell

**Figure:**
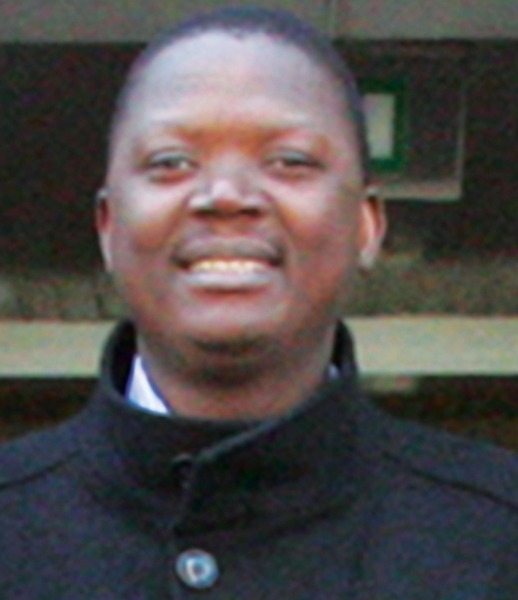
Nimzing G Ladep

**Figure:**
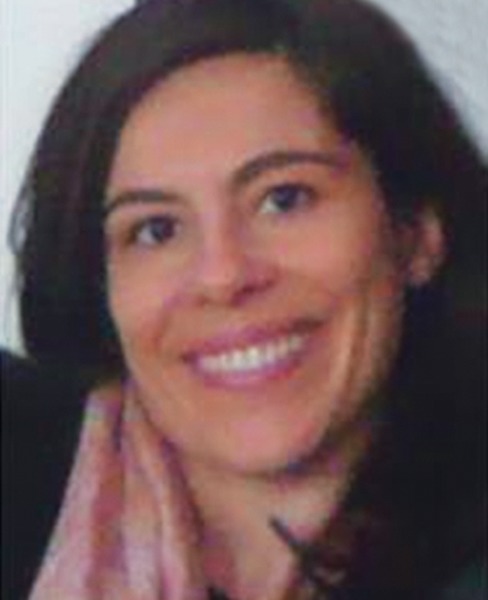
Maud Lemoine

The severity of the burden of HBV-related liver disease and liver cancer, coupled with the sheer scale of HBV prevalence worldwide, combines to make the HBV epidemic of critical importance to global public health (Available at: http://www.who.int/csr/disease/hepatitis/hepatitisB_whocdscsrlyo2002_2.pdf; Accessed on 21st April 2014).

The WHO has recommended universal HBV vaccination commencing within 24 hours of birth since 1992, in highly HBV-endemic countries,^2^ expanding this recommendation to all countries by 1997.^3^ By the end of 2012, 181 countries had implemented HBV vaccination through the Expanded Program for Immunization (EPI) and vaccination coverage was estimated to be 79% worldwide and 70% in Africa and South-Eastern Asia (Available at: www.who.int/mediacentre/factsheets/fs378/en/; Accessed on 21 Apr 2014). While HBV vaccination is critical for reducing HBV prevalence worldwide, estimates of the global impact of HBV vaccination on HBsAg prevalence predict a reduction in disease burden of 84%,^4^ demonstrating that other strategies to control and prevent infection are necessary.^5^ Real-world data from the Global Alliance for Vaccines and Immunization (GAVI) Alliance HBV vaccination program in China demonstrate a significant reduction in HBsAg prevalence in Chinese infants under 5 years of age from 9.7% (in 1992) to 0.96% (in 2013); however, 93 million people in China still remain infected.^6,7^

Accessible HBV treatment is a key for reducing the current global burden of HBV-related liver disease and liver cancer, and also helps in preventing transmission through lowering HBV viral load and, therefore, infectivity.^1,8^ Safe and effective medicines are widely available tor HBV infection, with tenotovir available at generic price in all continents through human immunodeficiency virus (HIV) treatment programs.^9^ However, these drugs remain inaccessible to the vast majority of HBV-infected patients worldwide. In many countries, patients can only access these medications if they are coinfected with HIV.^9,10^

The recent WHO (Global Policy Report on the Prevention and Control of Viral Hepatitis included data from a survey sent to all nations, to which 126 countries (64.9%) responded. Responding countries varied from 47.4% in the WHO-AFRO region to 100% in the WHO South-East Asia region. This included a spectrum of resource-replete and resource-poor countries. This survey revealed that of the responding countries, only 50.8% had clinical treatment guidelines for HBV and only 62.7% have publicly-funded HBV treatment available. A total of 81.7% have at least one HBV medication on their essential medicines list. However, it must be noted that tenofovir and entecavir, the two most effective nucleoside analogs with lowest barrier of resistance which are now recommended as first-line therapy,^1^ were only available in 48.4 and 34.9% of countries respectively. Considering the WHO regions with the highest endemicity of HBsAg prevalence (greater than 8%), publicly-funded HBV treatment was available in only 16.7% of WHO-AFRO countries, 54.3% of South-East Asian countries, and 53.3% of WPRO countries surveyed. (Available at: http://www.who.int/csr/disease/hepatitis/global_report/en/; Accessed on 21st April 2014).

**Figure:**
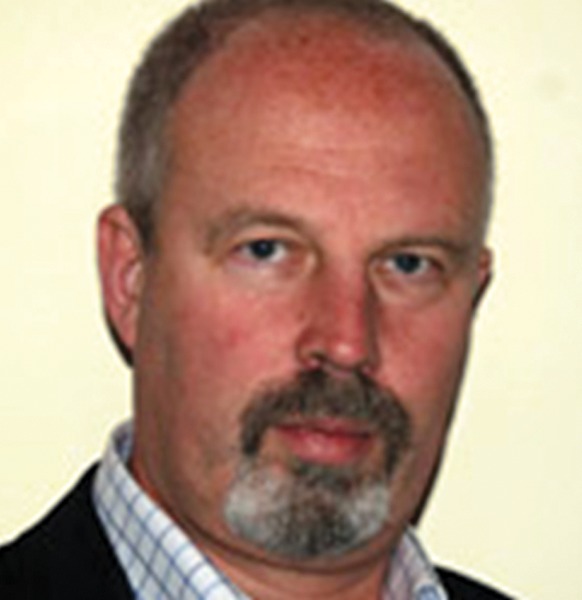
Mark R Thursz

**Figure:**
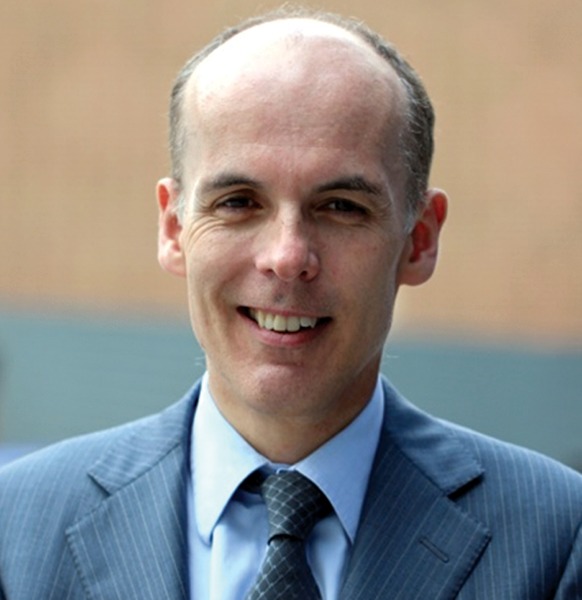
Simon D Taylor-Robinson

This staggering lack of accessibility to affordable, effective HBV treatments, despite availability of some of these agents for treatment of HIV, needs urgent amendment if any gains are to be made in preventing and controlling the burgeoning global HBV epidemic.

In 2010, the World Health Assembly (WHA) passed a resolution calling for public health intervention to prevent and control viral hepatitis. There is also a forthcoming WHA resolution, requesting the Global Health Fund to provide antiviral medications for HBV mono-infected patients. HBV treatment that is accessible and affordable to all is urgently needed. However, far greater pressure from the international medical community is required to build momentum to move political will and enhance support from the pharmaceutical industry. A concerted effort from international medical associations for liver disease and infectious diseases coupled with community hepatitis groups and healthcare workers is needed to educate and promote viral hepatitis among all levels of the global community. Only through working together, we will build international support for further vital research in viral hepatitis to inform governments and pharmaceutical companies, and help to enforce policy change.
